# Selenoprotein GPX3 regulates NADPH oxidase expression by inhibiting the MAPK signaling pathway and thereby attenuating the inflammatory response in renal ischemia-reperfusion injury

**DOI:** 10.1016/j.gendis.2025.101640

**Published:** 2025-04-11

**Authors:** Jun Pei, Jie Zhang, Chengjun Yu, Jin Luo, Sheng Wen, Yi Hua, Guanghui Wei

**Affiliations:** a Department of Urology, Children's Hospital of Chongqing Medical University, Chongqing 400014, China; b Ministry of Education Key Laboratory of Child Development and Disorders, Chongqing Key Laboratory of Pediatrics, National Clinical Research Center for Child Health and Disorders, China International Science and Technology Cooperation Base of Child Development and Critical Disorders, Children's Hospital of Chongqing Medical University, Chongqing 400014, China; c Chongqing Key Laboratory of Children Urogenital Development and Tissue Engineering, Chongqing 400014, China

**Keywords:** GPX3, Inflammation, Ischemia-reperfusion injury, Kidney, Selenoprotein

## Abstract

Renal ischemia-reperfusion injury (IRI) is one of the major causes of acute kidney injury, and the inflammatory response is considered a key factor. The selenoprotein GPX3, a member of the glutathione peroxidase family, has gradually attracted attention for its anti-inflammatory properties. However, the relationship between GPX3 and the inflammatory response during renal IRI remains unclear. The present study aims to investigate the role of GPX3 on the inflammatory response during renal IRI and related mechanisms. We utilized classic rat models of kidney IRI and cellular hypoxia reoxygenation model. After overexpressing GPX3 via lentiviruses and adeno-associated viruses, we observed a significant reduction in the expression levels of inflammatory factors in renal tissues, along with an increase in the expression of anti-inflammatory factor IL-10, resulting in noticeable alleviation of renal IRI. Meanwhile, we found that GPX3 alleviated the inflammatory response, probably by inhibiting the MAPK signaling pathway and reducing the expression of NAPDH oxidase. To further validate the mechanism by which GPX3 alleviated the inflammatory response, we used the MAPK signaling pathway agonist anisomycin for intervention. The results showed that anisomycin intervention significantly reversed the inhibitory effect of GPX3 on the MAPK signaling pathway, in which the expression level of NADPH oxidase was significantly increased, the secretion of inflammatory factors was increased, and the degree of renal tissue damage was significantly increased. These findings suggest that selenoprotein GPX3 alleviates inflammation during renal IRI by inhibiting the MAPK signaling pathway and reducing NADPH oxidase expression.

## Introduction

Acute kidney injury (AKI) is characterized by a rapid decrease in glomerular filtration rate over a short period, resulting in impaired kidney function, elevated serum creatinine levels, and even symptoms such as oliguria or anuria. Some patients may require renal replacement therapy, and severe cases can lead to death.^1^ AKI can be triggered by trauma, sepsis, surgery, or nephrotoxic drugs, with ischemia-reperfusion injury (IRI) being one of the common causes.^2^ Renal IRI disrupts the redox balance of cells, leading to excessive production of reactive oxygen species (ROS) in renal tissue. This induces a series of events, including mitochondrial dysfunction, enhanced microvascular permeability, release of numerous inflammatory factors, and apoptosis and necrosis of renal tubules.^3^^,^^4^ In addition, incomplete recovery from IRI-induced AKI can lead to renal fibrosis, thereby increasing the risk of chronic kidney disease and end-stage renal failure.^5^, ^6^, ^7^ Therefore, effectively preventing or mitigating renal IRI is crucial for the recovery of renal function following ischemic events.

Renal IRI can trigger an inflammatory response through various mechanisms, such as ROS release, immune system activation, cytokine secretion, and iron death.^8^, ^9^, ^10^ The inflammatory response plays a crucial role in the pathophysiology of AKI. During AKI, the expression of inflammation-related biomarkers increases significantly, leading to damage to renal tissue. Several studies have shown that blocking the inflammatory response alleviates the associated AKI phenotype.^11^^,^^12^ Thus, inhibiting inflammation is one of the key strategies to mitigate renal IRI.

Selenium is an essential trace element that is present in proteins (*i.e.*, selenoproteins) in the form of selenocysteine (Sec).^13^ To date, 25 selenoprotein genes have been identified in the human genome, including glutathione peroxidase (GPX), thioredoxin reductase, iodothyronine deiodinases, and other selenoproteins.^14^ They are essential for maintaining human development and health.^15^ Research has shown that selenoproteins play an important regulatory role in inflammation by suppressing the expression of inflammatory factors and thereby alleviating tissue and organ damage.^16^ The selenoprotein glutathione peroxidase 3 (GPX3) is a member of the GPX family, is primarily secreted by renal tubular epithelial cells and then released into the bloodstream.^17^ GPX3 is the only selenoprotein of the GPX family that can be detected in plasma and is considered the first barrier involved in cellular redox homeostasis. However, the relationship between GPX3 and the inflammatory response during renal IRI, as well as the mechanisms by which it alleviates inflammation, has not yet been reported.

In this study, we utilized the classical rat renal IRI model and cellular hypoxia reoxygenation (H/R) model to comprehensively explore the specific mechanism of action of selenoprotein GPX3 to alleviate the inflammatory response during renal IRI, providing a theoretical basis for its clinical application in treating renal IRI.

## Materials and methods

### Materials and reagents

The primary antibodies used in this study were sourced as follows: anti-GPX3 (abcepta, Cat#AP11221c), anti-NGAL (ABclonal, Cat#A2092), anti-BAX (ABclonal, Cat#A0207), anti-NOX2 (ABclonal, Cat#A1636), anti-NOX4 (ABclonal, Cat#A23465 and Cat#A22149), anti-p22^phox^ (ABclonal, Cat#A10694), anti-TNF-α (ABclonal, Cat#A11534), anti-IL-6 (Abcam, Cat#ab290735), anti-IL1-β (Abcam, Cat#ab315084), anti-IL-10 (ABclonal, Cat#A2171), anti-P38 (ABclonal, Cat#A14401), anti-p-P38 (ABclonal, Cat#APs0057), anti-JNK (Proteintech, Cat#28007-1-AP), and anti-p-JNK (ABclonal, Cat#AP0631). Anisomycin (Cat#HY-18982), a MAPK pathway activator, and dihydroethidium (Cat#HY-D0079) were purchased from MCE. TUNEL Andy Fluor™ 488 Apoptosis Detection Kit was purchased from ABP Biosciences (Cat#A050).

### Animal experiments

All animal experiments were approved by the Animal Experimental Ethics Committee of the Children’s Hospital of Chongqing Medical University and followed the ARRIVE guidelines. Adult male Sprague–Dawley rats, weighing 250–280 g, were purchased from the Experimental Animal Center of Chongqing Medical University. The animals were housed under standardized conditions with controlled temperature and humidity and a 12-h/12-h light/dark cycle and were provided with standard food and water *ad libitum*. The rats were randomly divided into four groups, with five rats in each group: Sham, IRI, Sham + GPX3, and IRI + GPX3. The procedure for constructing the renal IRI model was as follows. After routine anesthesia, hair removal, disinfection, and draping, the right kidney was first removed. The left renal pedicle was bluntly separated, and a non-invasive vascular clamp was used to occlude the left renal pedicle for 45 min. Successful ischemia was indicated by the kidney color changing from bright red to purplish-black. The clamp was then released, and successful reperfusion was indicated by the kidney color gradually returning to bright red. In the Sham group, the left renal pedicle was only bluntly separated without inducing ischemia. After 24 h, rats were killed suddenly under anesthesia, and blood was taken from the inferior vena cava to test the renal function of the rats; the left kidney was taken, half of which was quickly placed in a −80 °C refrigerator for storage, and the other half of which was fixed using 4% paraformaldehyde.

### Cell culture and H/R model establishment

In the cell experiments, the NRK-52E cell line was used as the research subject. All cells were cultured in Dulbecco's modified Eagle medium containing 10% fetal bovine serum and 1% penicillin/streptomycin. The cell experiments employed a classical H/R model to simulate the process of renal IRI. The specific procedure was as follows. First, we cultured and pretreated the cells under normoxic conditions (21% O_2_, 5% CO_2_, 37 °C), and after the cells grew to 60%–70%, they were replaced with 1% glucose Dulbecco's modified Eagle medium. Next, the cells were incubated anoxically in a three-gas incubator (Thermo Scientific, Waltham, MA, USA) for 6 h (1% O_2_, 5% CO_2_, 37 °C), and then the conventional medium was replaced, and incubation was continued for 24 h under normoxic conditions. Anisomycin, an effective MAPK pathway activator,^18^^,^^19^ was used to activate the MAPK pathway at a concentration of 5 μM.

### Adeno-associated virus (AAV)-mediated overexpression in rats

The overexpression AAV (GPX3) was synthesized by GeneChem Co., Ltd. in Shanghai (Cat#10061222). GPX3 AAV (AAV9-GPX3; 1.09E+13 viral genome copies per milliliter; promoter: cytomegalovirus) was injected via the tail vein (diluted using 9 mL saline; total amount injected per rat: 1.09E+11 viral genome copies (100 μL)). The control group received an injection of an empty vector via the tail vein. The procedure was strictly carried out according to the manufacturer's instructions. Two weeks later, the rats were anesthetized and subjected to the renal IRI model as previously described.

### Lentiviral overexpression in cells

The culture conditions for NRK-52E cells were the same as described above. The overexpression lentivirus (LV-GPX3; 1.5E+9 transducing units per milliliter; vector: GV513; element order: Ubi-MCS-CBh-gcGFP-IRES-puromycin) was synthesized by GeneChem Co., Ltd. in Shanghai (Cat#10027761). We cultured the cells in a 96-well plate and divided them into five groups based on different multiplicity-of-infection (MOI) values (MOI 10, MOI 20, MOI 30, and MOI 50). The cells were incubated in a 37 °C incubator with 5% CO_2_ for 24 h. After removing the medium containing the lentivirus, the cells were further cultured in fresh medium for 72 h, with the medium being changed every 24 h. The transfection efficiency of the lentivirus at different MOI values was observed under a fluorescence microscope to determine the optimal MOI value. The procedure was strictly carried out according to the manufacturer's instructions. Subsequent cell experiments were conducted using the established cell models under the determined optimal MOI conditions.

### Rat serum assay

About 3 mL of blood was taken from the inferior vena cava of rats under anesthesia, and the upper layer of serum was centrifuged and placed in an automatic biochemical analyzer (Cobas C701, Roche, Basel, Switzerland) to detect the levels of urea nitrogen and creatinine in each group, which were used to assess the renal impairment of the rats in each group. Serum expression levels of aspartate aminotransferase (AST) and alanine aminotransferase (ALT) were also examined to assess preliminarily whether the intervention of AAV9-GPX3 caused damage to liver function.

### Hematoxylin-eosin staining and immunohistochemistry staining

Kidney tissues were fixed in 4% paraformaldehyde for 24 h, dehydrated, embedded, and sectioned into 4 μm slices. The sections were deparaffinized and rehydrated according to standard procedures, followed by hematoxylin-eosin staining and immunohistochemistry staining. The immunohistochemistry staining procedure was as follows. Antigen retrieval was performed by heating the sections in a citrate buffer. After quenching endogenous peroxidase activity and blocking with bovine serum albumin, the sections were washed with phosphate buffer saline and incubated with primary antibodies at 4 °C overnight (all primary antibodies were diluted at 1:200). The following day, the sections were washed with phosphate buffer saline, incubated with a reaction enhancer, followed by secondary antibodies, and then counterstained with hematoxylin. Finally, the sections were dehydrated through an alcohol gradient, cleared with xylene, and mounted.

### Immunofluorescence staining

NRK-52E cells were cultured on cell crawls in 24-well plates, routinely fixed with 4% paraformaldehyde, permeabilized with Triton, blocked with bovine serum albumin and washed with phosphate buffer saline, incubated with primary antibody, and placed in the refrigerator at 4 °C overnight. On the second day, the cell slides were incubated with secondary antibody at room temperature for 1 h in the dark and then washed routinely with phosphate buffer saline; stained with phalloidin in the dark for 1 h and then washed again with phosphate buffer saline; stained with DAPI in the dark for 10 min; and then blocked and visualized under the A1R confocal microscopy system (Nikon, Japan).

### TUNEL assay

NRK-52E cells were cultured on cell crawls according to the different treatment conditions described above, routinely fixed in 4% paraformaldehyde, permeabilized with Triton, and then subjected to TUNEL according to the kit instructions. Immunofluorescence images were taken and analyzed by an A1R confocal microscopy.

### Measurement of intracellular ROS in cells

ROS levels in cells were measured with dihydroethidium (MCE, HY-D0079) according to its use instructions. The cells were cultured on cell crawls according to the experimental design, and then the medium was aspirated and discarded. After that, 500 μL of dihydroethidium at a final concentration of 10 μmol/L was added to each well, and the cells were incubated in a 37 °C incubator with 5% CO_2_ in the dark for 30 min. The cells were then washed three times with a serum-free medium and observed under a fluorescence microscope.

### Western blotting

Proteins were extracted from rat kidney tissues and NRK-52E cells using RIPA lysis buffer containing protease inhibitors, and protein concentrations were determined using a bicinchoninic acid kit (Beyotime, Cat#P0012). Equal amounts of protein samples were separated by SDS-PAGE and then transferred onto PVDF membranes. After blocking, the membranes were incubated with primary antibodies (diluted to a concentration of 1:1000) at 4 °C overnight. The next day, after washing with tris-buffered saline containing 0.1% Tween 20, the membranes were incubated with secondary antibodies (diluted to a concentration of 1:15,000) at room temperature for 1 h. Protein bands were visualized using the ChemiDoc System (Bio-Rad, Hercules, CA, USA). Finally, a semi-quantitative analysis of the Western blot bands was performed using ImageJ software.

### Statistical analysis

Statistical analysis of experimental data was conducted using Image J and Prism software (GraphPad Software, La Jolla, CA). Differences between two groups were assessed using the unpaired student's *t*-test. One-way analysis of variance (ANOVA) was employed for comparisons among multiple groups. *p*-values < 0.05 were considered statistically significant. The symbols used for indicating significance levels are as follows: ∗∗∗∗*p* < 0.0001, ∗∗∗*p* < 0.001, ∗∗*p* < 0.01, and ∗*p* < 0.05.

## Results

### Effect of selenoprotein GPX3 on IRI in rat kidney

**Figure 1:**
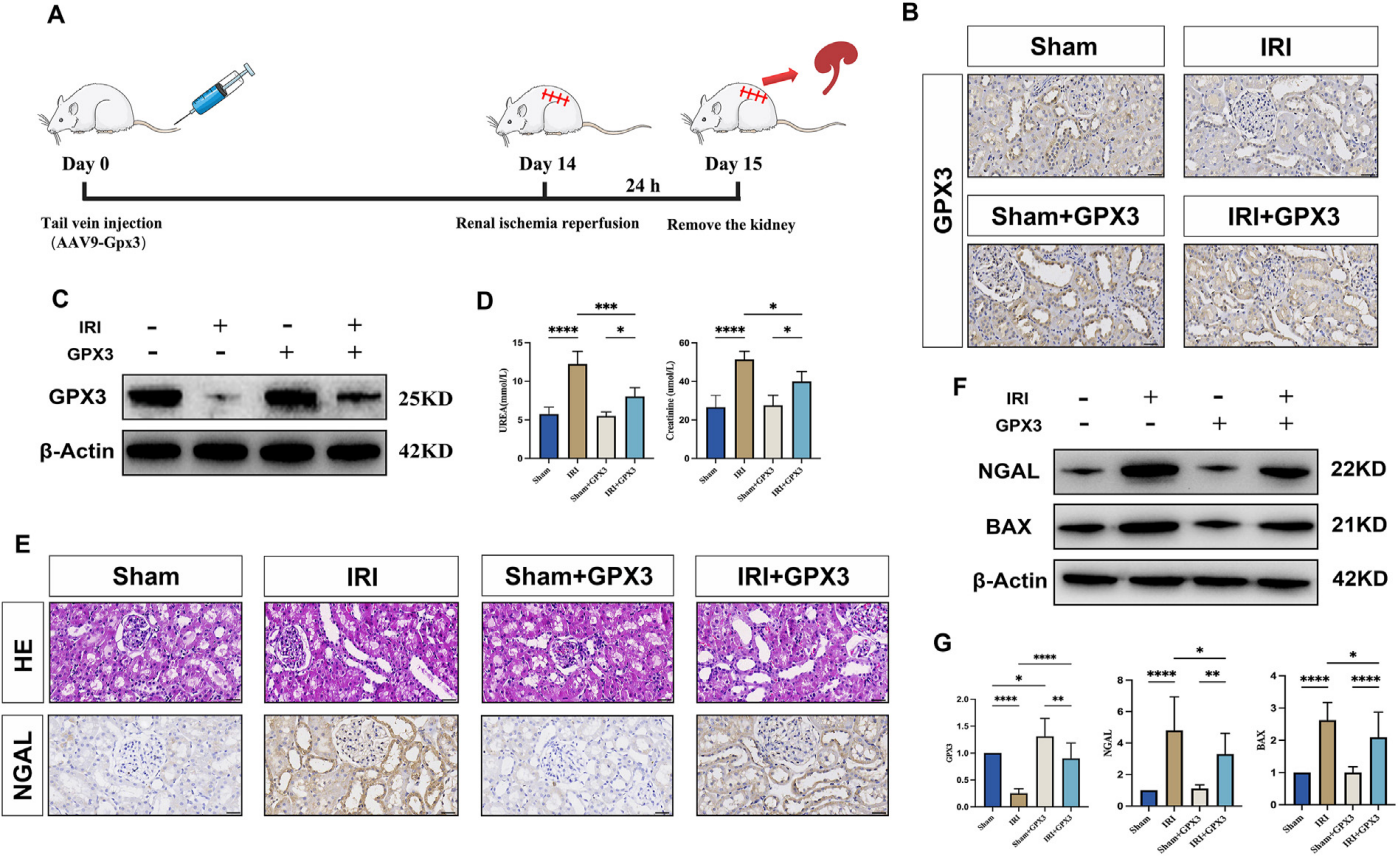
Overexpression of GPX3 alleviates ischemia-reperfusion injury. **(A)** The experimental process in animals. **(B)** Immunohistochemistry-stained GPX3 expression levels in rat kidney tissues from each group. **(C)** Western blot bands of GPX3 expression levels in rat kidney tissues from each group. **(D)** A statistical plot of the expression levels of urea nitrogen and creatinine in the serum of rats in each group. **(E)** Hematoxylin-eosin-stained and immunohistochemistry-stained NGAL expression levels in the kidney tissues of rats in each group. **(F)** Western blot bands of NGAL and BAX expression levels in rat kidney tissues from each group. **(G)** Western blot band statistics.

In the present study, we verified the effect of GPX3 on renal IRI after overexpression of GPX3 in animal experiments by injecting GPX3 AAV through the tail vein of rats (Fig. 1A). First, we verified the expression level of GPX3 in rat kidney tissues by immunohistochemistry and western blotting. We found that the expression level of GPX3 was significantly increased in the Sham + GPX3 group compared with the Sham group (Fig. 1B, C, G) and similarly significantly increased in the IRI + GPX3 group compared with the IRI group (Fig. 1B, C, G). This confirms the success of our modeling. Meanwhile, we found by immunohistochemistry that GPX3 was mainly located in renal tubules for expression (Fig. 1B), which was caused by the fact that GPX3 was mainly secreted by renal tubular epithelial cells. Next, we found that when subjected to IRI, the expression level of GPX3 was significantly reduced (Fig. 1B, C, G), renal function was impaired (Fig. 1D), renal histopathological damage was aggravated (Fig. 1E), and the expression levels of renal tubular epithelial cell damage marker (NGAL) and apoptotic protein (BAX) were significantly increased (Fig. 1E–G). After being subjected to GPX3 overexpression, serum levels of urea nitrogen and creatinine were significantly reduced (Fig. 1D), renal histopathological damage was significantly reduced, and the expression levels of NGAL and BAX in renal tissues were significantly reduced compared with those in the IRI group (Fig. 1E–G). GPX3 was demonstrated to be effective in alleviating renal IRI.

### Effect of selenoprotein GPX3 on inflammatory response in IRI in rat kidney

**Figure 2:**
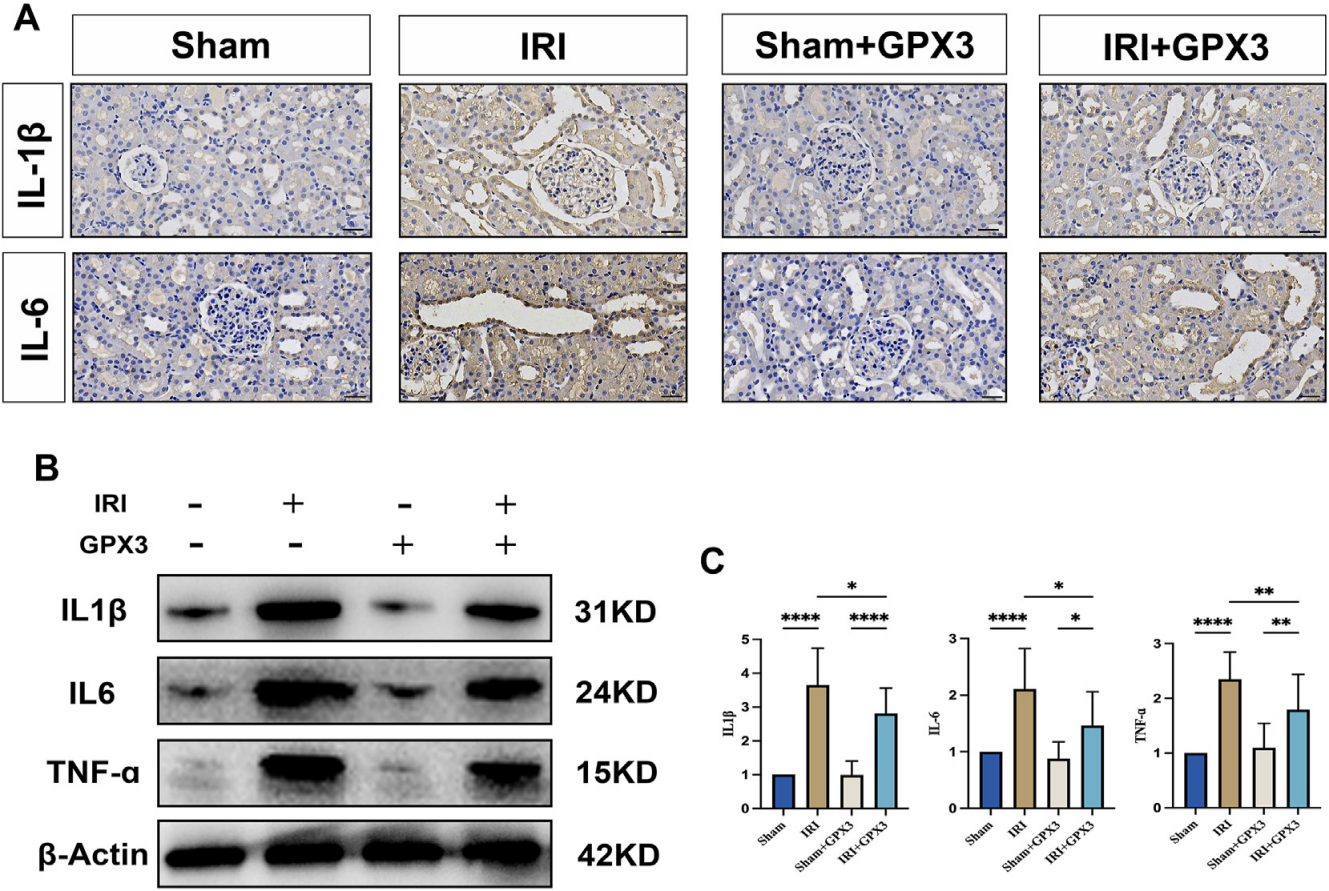
GPX3 overexpression inhibits the inflammatory response during renal ischemia-reperfusion injury. **(A)** Immunohistochemistry staining images of the expression levels of inflammatory factors (IL1β and IL-6) in renal tissues of each group of rats. **(B, C)** Western blot band images and statistical graphs of the expression levels of inflammatory factors (IL1β, IL-6, and TNF-α) in renal tissues of each group of rats.

In the present study, after the detection of inflammatory factors (IL1-1β, TNF-ɑ, and IL-6), it was found that the expression of the inflammatory factors was significantly increased in the IRI group compared with the Sham group (Fig. 2A–C). However, after GPX3 overexpression, the expression levels of the inflammatory factors were significantly reduced (Fig. 2A–C). This implies that GPX3 can inhibit the expression level of inflammatory factors and alleviate the inflammatory response during renal IRI.

### Effects of selenoprotein GPX3 on the MAPK signaling pathway

**Figure 3:**
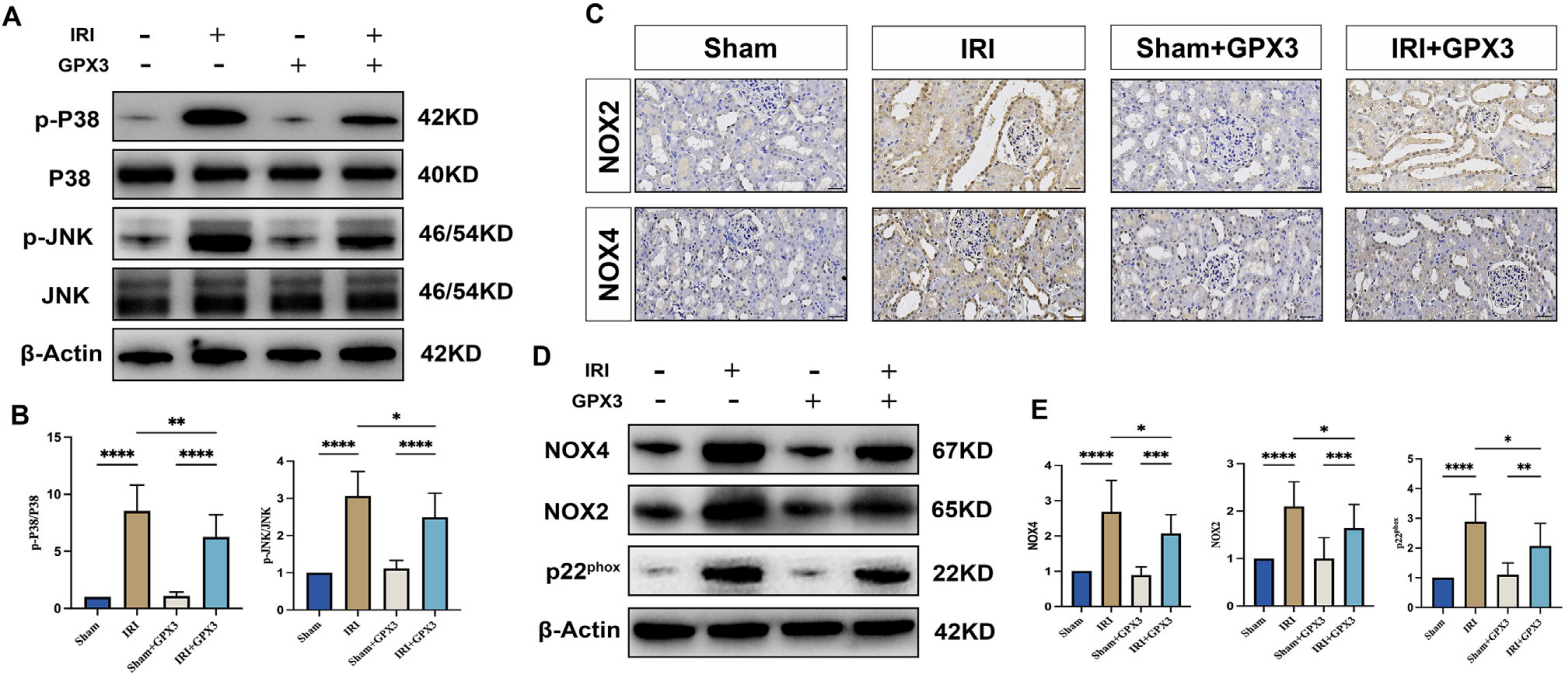
GPX3 overexpression inhibits the MAPK signaling pathway and NADPH oxidase. **(A, B)** Western blot band charts and statistical graphs of the expression levels of MAPK signaling pathway in the kidney tissues of rats in each group. **(C)** The immunohistochemical plots of NADPH oxidase (NOX2 and NOX4) expression levels in the kidney tissues of rats in each group. **(D)** Western blot band plots of NADPH oxidase (NOX4, NOX2, and p22^phox^) expression levels in kidney tissues of rats from each group. **(E)** A statistical plot of Western blot bands.

In our previous study, we found that inhibition of the MAPK signaling pathway was effective in alleviating renal IRI.^20^ To further clarify whether GPX3 played a protective role by inhibiting the MAPK signaling pathway during IRI in rat kidney, we used western blotting to detect the key proteins of the MAPK signaling pathway again. We found that the expression of key proteins of the MAPK signaling pathway was significantly higher in the IRI group compared with the Sham group (Fig. 3A, B), demonstrating that the MAPK signaling pathway is significantly activated when the kidney is subjected to IRI. After overexpression of GPX3, the expression levels of key proteins of the MAPK signaling pathway were significantly reduced compared with the IRI group (Fig. 3A, B), demonstrating the ability of GPX3 to inhibit the MAPK signaling pathway in the rat renal IRI model.

### Effect of selenoprotein GPX3 on NADPH oxidase (NOX)

Previous studies have found a close link between the expression level of NADPH oxidase (NOX) and the MAPK signaling pathway.^21^ NOX is not only a major source of ROS but likewise a key regulator of the inflammatory response.^22^ In renal tissues, NOX2 and NOX4 are expressed at the highest levels.^23^ NOX proteins do not have catalytic activity on their own, they need to bind to each other with a variety of regulatory subunits to form stable complexes before they can function, of which p22^phox^ is the most common stabilizing factor of NOX complexes.^23^ To further explore the effect of selenoprotein GPX3 on NOX oxidase, we found that the expression levels of NOX2, NOX4, and p22^phox^ were significantly increased in the renal tissues of rats in the IRI group compared with the Sham group (Fig. 3C–E), which implies that renal IRI was able to induce NOX oxidase expression. When subjected to the intervention of selenoprotein GPX3, the expression levels of NOX2, NOX4, and p22^phox^ were significantly reduced (Fig. 3C–E), demonstrating the ability of selenoprotein GPX3 to inhibit the expression of NOX oxidase.

### Effect of selenoprotein GPX3 on the level of cell damage and apoptosis

**Figure 4:**
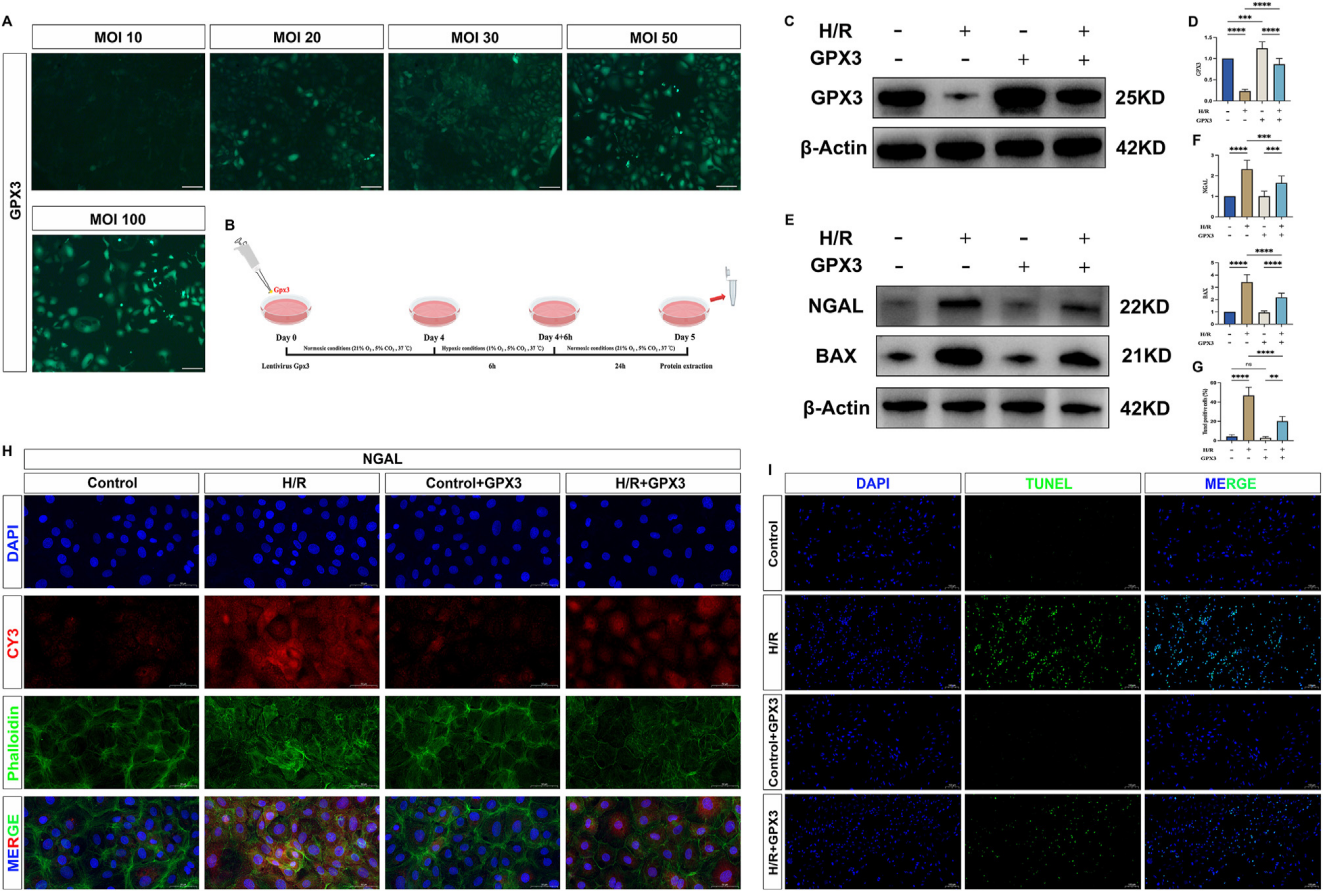
Overexpression of GPX3 alleviates cell damage. **(A)** Fluorescence images of transfection efficiency of lentiviral GPX3 at different concentrations (MOI 10, MOI 20, MOI 30, MOI 50, and MOI 100) in cells. **(B)** Schematic diagram of the cell experiment. **(C, D)** Western blot band plots and statistical plots of GPX3 expression levels in each group of cells under the condition of MOI 50. **(E, F)** Western blot band plots and statistical graphs of the expression levels of the apoptotic protein BAX and the renal tubular injury marker NGAL in the cells of each group. **(G)** Statistical graph of apoptosis levels detected by TUNEL. The *Y*-axis represents the ratio of TUNEL-positive cells to normal cells. **(H)** Fluorescence plot of the expression level of the renal tubular injury marker NGAL in each group of cells. **(I)** The level of apoptosis in each group of cells using TUNEL. The green represents TUNEL-positive cells, and the blue represents nuclei.

To further elucidate the role of GPX3, we utilized a cell H/R model in rat NRK-52E cells to simulate kidney IRI, and concurrently employed lentiviral overexpression of GPX3 to explore its potential mechanisms (Fig. 4B). First, we found that the infection efficiency of GPX3 in cells was positively correlated with MOI, which gradually increased with the increase of MOI value. Among them, lentiviral GPX3 was able to be significantly overexpressed in rat NRK-52E cells when the MOI value was 50. Considering that too high MOI would affect the state of cell growth, we used this concentration for subsequent studies (Fig. 4A). Furthermore, compared with the Control group, GPX3 expression was significantly decreased in the H/R group (Fig. 4C, D). At this stage, the expression levels of NGAL and BAX were significantly increased (Fig. 4E, F, H). TUNEL assay likewise confirmed significantly higher levels of apoptosis (Fig. 4G–I). Upon GPX3 overexpression, the expression levels of NGAL and BAX in the H/R + GPX3 group were decreased compared with the H/R group (Fig. 4E, F, H), and apoptosis levels were also significantly reduced (Fig. 4G–I). This demonstrates that the overexpression of GPX3 can alleviate cell damage and apoptosis.

### Effect of selenoprotein GPX3 on MAPK signaling pathway and NOX

**Figure 5:**
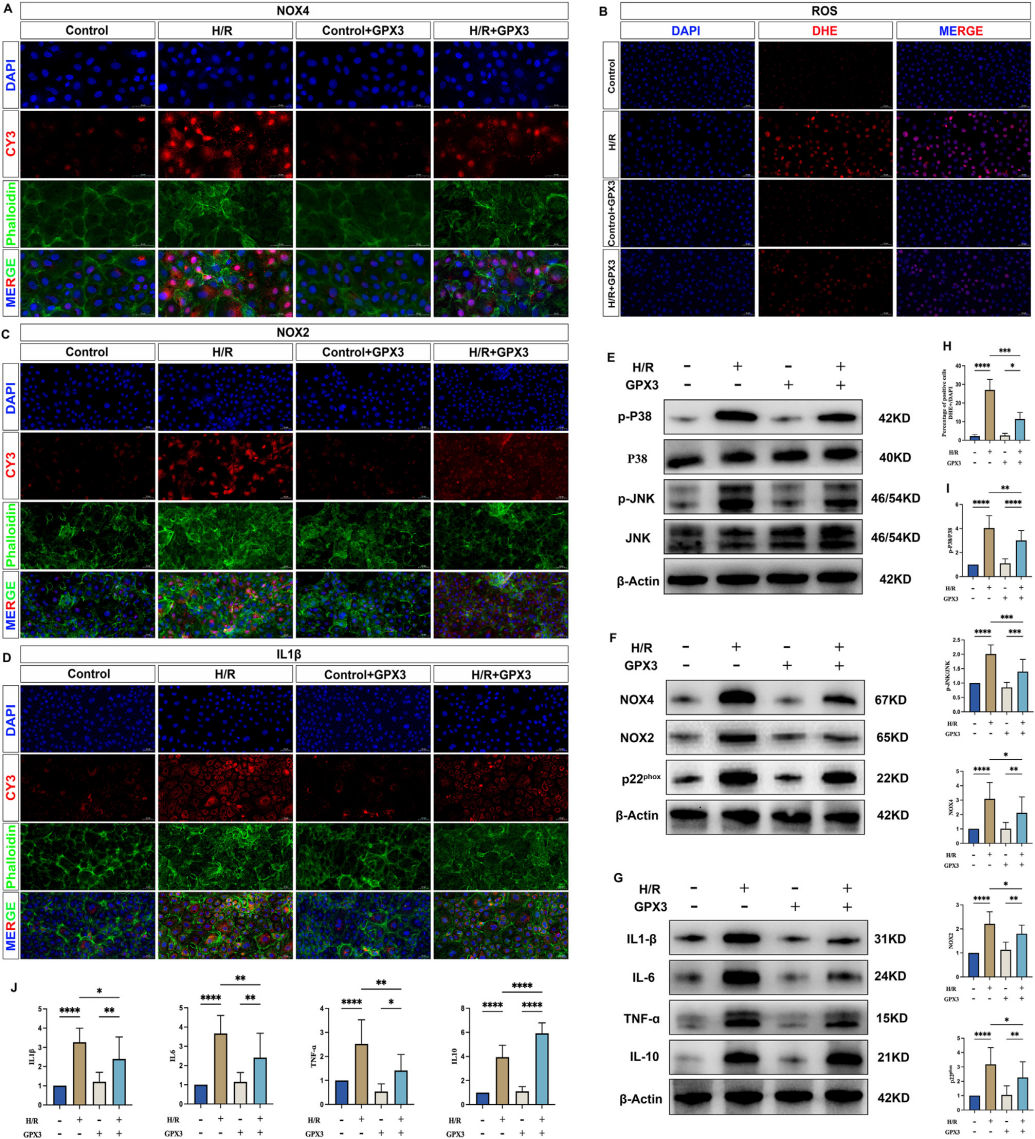
Mechanism of action of GPX3 overexpression. **(A)** Fluorescence plot of NOX4 expression levels in each group of cells. **(B)** Fluorescent plots of ROS expression levels in each group of cells. **(C)** Fluorescence plot of NOX2 expression levels in each group of cells. **(D)** Fluorescent plots of IL1β expression levels in each group of cells. **(E)** Western blot band plots of the expression levels of key proteins of the MAPK signaling pathway in each group of cells. **(F)** Western blot band plots of the expression levels of key NADPH oxidase proteins in each group of cells. **(G)** Western blot band plots of the expression levels of inflammatory and anti-inflammatory factors. **(H)** Statistical plot of reactive oxygen species (ROS) expression levels. **(I, J)** Statistical plots of Western blot bands.

We re-validated the role of selenoprotein GPX3 on the MAPK signaling pathway and NOX in a cellular H/R model. We found that the MAPK signaling pathway was significantly activated (Fig. 5E–I), the expression level of NOX oxidase was significantly increased (Fig. 5A–C, F, I), and the expression of ROS was significantly increased (Fig. 5B–H) in the H/R group compared with the Control group, which was in keeping with the results of the animal experiments. However, upon GPX3 overexpression, the MAPK signaling pathway was significantly inhibited compared with the H/R group (Fig. 5E–I), the expression level of NOX was reduced (Fig. 5A–C, F, I), and the secretion of ROS was significantly reduced (Fig. 5E–I). Once again, GPX3 was shown to inhibit the MAPK signaling pathway and reduce NOX oxidase and ROS production.

### Effect of selenoprotein GPX3 on inflammatory response

In our study, we found that the expression levels of inflammatory factors (IL1β, IL6, and TNF-ɑ) and anti-inflammatory factor (IL10) were significantly higher in the H/R group, compared with the Control group (Fig. 5D–G, J), implying that the level of inflammation in the cells was significantly increased when the cells were subjected to H/R, which was in keeping with the results of animal experiments. The expression levels of inflammatory factors (IL1β, IL6, and TNF-ɑ) were significantly reduced, and the expression of anti-inflammatory factor (IL10) was significantly increased upon GPX3 overexpression, compared with the H/R group (Fig. 5D–G, J). This again demonstrates the ability of GPX3 to alleviate inflammatory levels in cells and increase the release of anti-inflammatory factors.

### Anisomycin reverses the protective effects of selenoprotein GPX3

**Figure 6:**
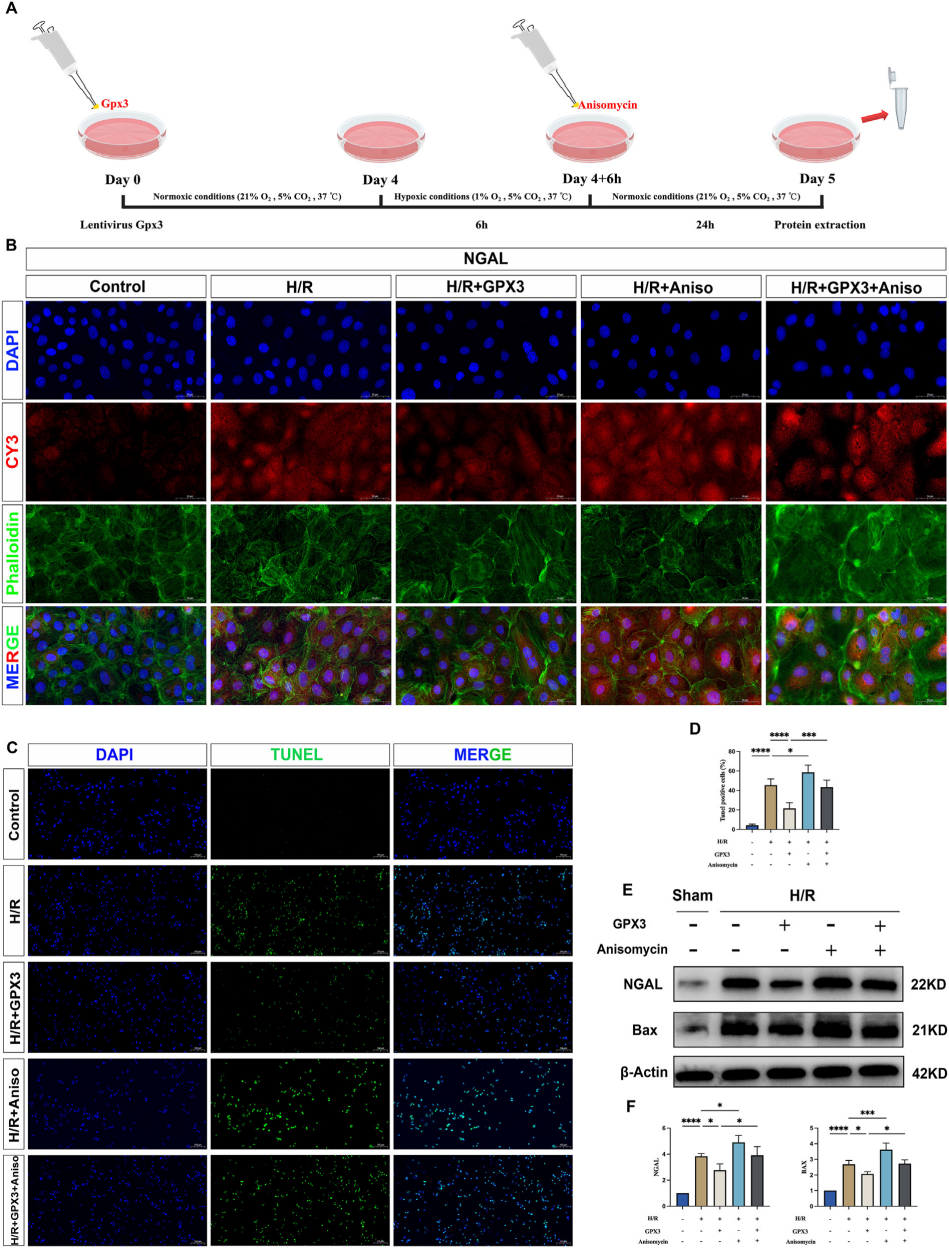
Anisomycin reverses the protective effect of selenoprotein GPX3. **(A)** The flow chart of the study. **(B)** Fluorescence plot of NGAL expression levels in each group of cells. **(C)** Fluorescence plot of apoptosis levels in each group using TUNEL. **(D)** Statistical graph of apoptosis levels detected by TUNEL. **(E)** Western blot band plots of NGAL and BAX expression levels in each group of cells. **(F)** Western blot band statistics.

To further demonstrate that selenoprotein GPX3 could regulate the expression of NOX oxidase by inhibiting the MAPK signaling pathway, thereby alleviating the inflammatory response, we activated the MAPK signaling pathway using anisomycin in rat NRK-52E cells (Fig. 6A). We found that the expression levels of NGAL and BAX were significantly increased in the H/R + AAnisomycin group compared with the H/R group (Fig. 6B–E, F). TUNEL assay likewise revealed that apoptotic levels of cells were significantly increased (Fig. 6C, D). This implies that the activation of the MAPK signaling pathway by anisomycin can further aggravate the level of cellular damage, which again suggests that the activation of the MAPK signaling pathway is closely linked to cellular damage. Next, we found that NGAL and BAX expression were significantly increased in the H/R + GPX3+Anisomycin group compared with the H/R + GPX3 group (Fig. 6B–E, F), and the level of apoptosis was significantly increased, as shown by TUNEL assay (Fig. 6C, D). This demonstrates that anisomycin, an agonist of the MAPK signaling pathway, reverses the protective effects of GPX3.

### Anisomycin reverses the inhibitory effect of selenoprotein GPX3 on the MAPK signaling pathway

**Figure 7:**
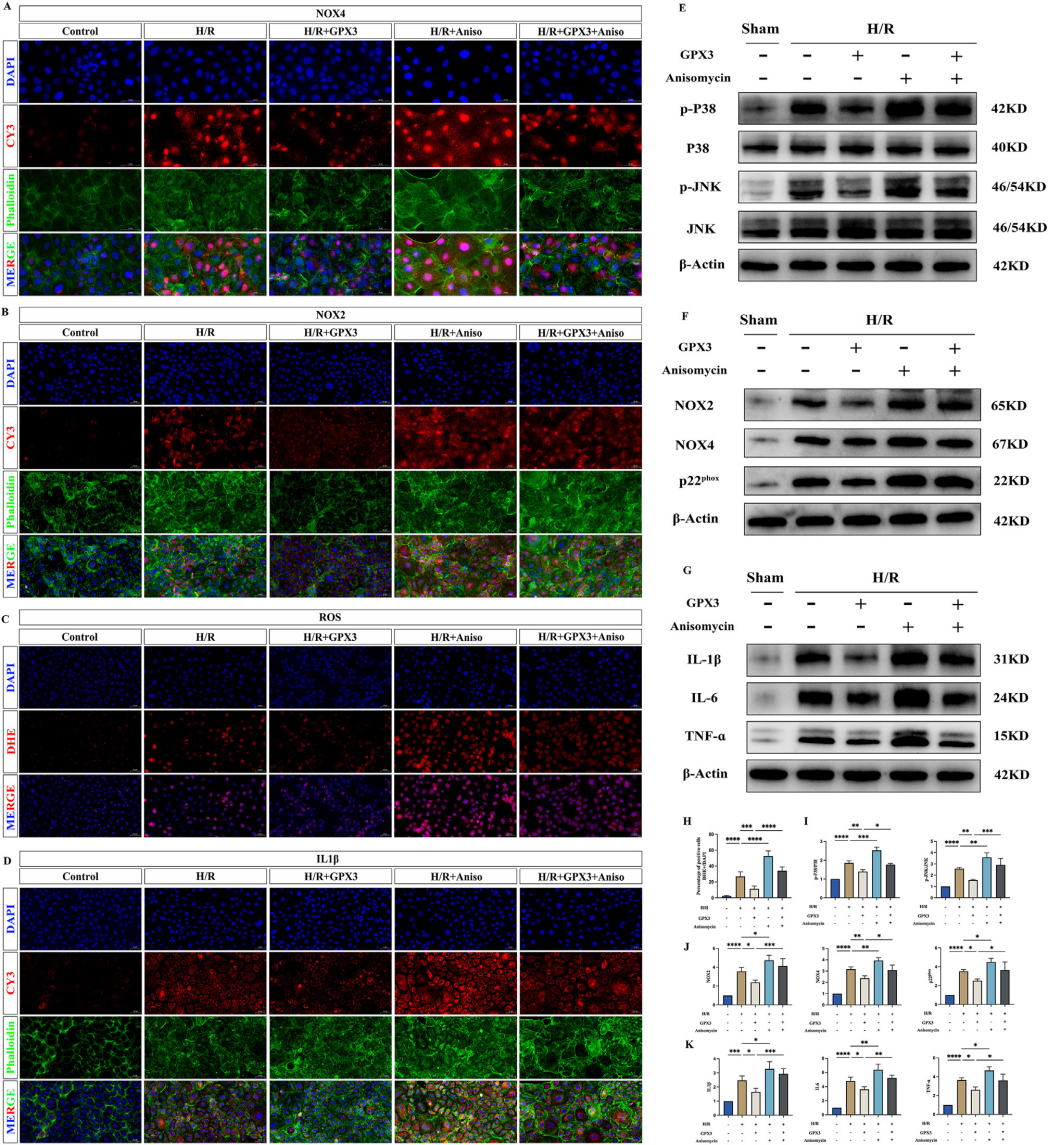
Validation of the mechanism of action of selenoprotein GPX3. **(A)** Fluorescence plot of NOX4 expression levels in each group of cells. **(B)** Fluorescent plots of NOX2 expression levels in each group of cells. **(C)** Fluorescence plot of ROS expression levels in each group of cells. **(D)** Fluorescent plots of IL1β expression levels in each group of cells. **(E)** Western blot band plot of the expression level of the MAPK signaling pathway. **(F)** Western blot band plots of NADPH oxidase (NOX2, NOX4, and p22^phox^) expression levels. **(G)** Western blot band plots of expression levels of inflammatory factors (IL1β, IL6, and TNF-ɑ). **(H)** Statistical plot of reactive oxygen species (ROS) expression levels. **(I)** Western blot band statistics of the MAPK signaling pathway. **(J)** Statistical plot of Western blot bands for NOX oxidase. **(K)** Statistical plot of Western blot bands for inflammatory factors.

The above results demonstrated that anisomycin intervention was able to reverse the protective effect of GPX3 on cells. To further verify that the protective effect of GPX3 was achieved by inhibiting the MAPK signaling pathway and regulating NOX oxidase secretion, we again examined the expression levels of the MAPK signaling pathway, NOX oxidase, ROS, and inflammatory factors. We found that the MAPK signaling pathway was further activated in the H/R + Anisomycin group compared with the H/R group, which has verified that anisomycin is an agonist that can effectively activate the MAPK signaling pathway (Fig. 7E–I). Meanwhile, the expression levels of NOX2, NOX4, and p22^phox^ were significantly increased (Fig. 7A, B, F, J), ROS production was significantly increased (Fig. 7C–H), and the expression of inflammatory factors (TNF-ɑ, IL1β, and IL6) was increased (Fig. 7D–G, K). This demonstrates that the activation of the MAPK signaling pathway can further promote the secretion of NOX oxidase, increase the expression level of ROS, and enhance the inflammatory response. Next, we found that the expression level of the MAPK signaling pathway was increased in the H/R + GPX3 + Anisomycin group compared with the H/R + GPX3 group, implying that anisomycin was able to reverse the inhibitory effect of GPX3 on the MAPK signaling pathway (Fig. 7E–I). Meanwhile, the expression levels of NOX2, NOX4, and p22^phox^ were further increased (Fig. 7A, B, F, J), promoting ROS production (Fig. 7C–H), and the expression of inflammatory factors (TNF-ɑ, IL1β, and IL6) was likewise significantly increased (Fig. 7D–G, K). This demonstrated that when anisomycin reversed the inhibitory effect of GPX3 on the MAPK signaling pathway, the expression of NOX oxidase was further increased, promoting ROS production and exacerbating the inflammatory response. Therefore, we suggest that the selenoprotein GPX3 may regulate the expression of NOX oxidase by inhibiting the MAPK signaling pathway, thereby alleviating the inflammatory response.

## Discussion

**Figure 8:**
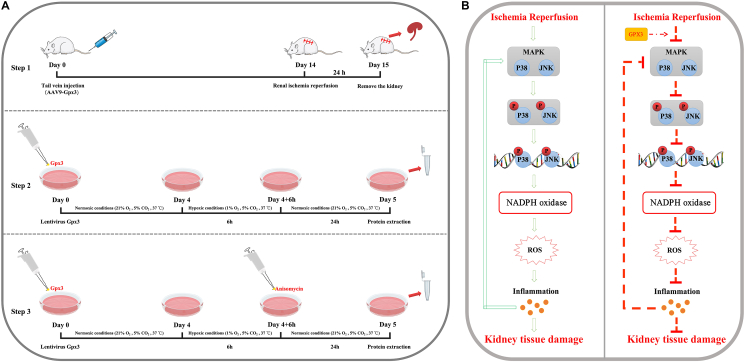
Flowchart and mechanism diagram for this study. **(A)** The overall process of this study. **(B)** The mechanism of interest for this study. The green arrows represent facilitation, and the red arrows represent inhibition.

This study aims to provide evidence on whether selenoprotein GPX3 can alleviate the inflammatory response during renal IRI and to propose possible mechanisms mediating this effect, demonstrating that GPX3 could be a novel therapeutic target for mitigating renal IRI. We constructed classic rat models of renal IRI and cell H/R models, and overexpressed GPX3 using adenoviral and lentiviral vectors both *in vivo* and *in vitro* to explore the mechanisms by which GPX3 inhibits inflammation (Fig. 8A). The results suggest that selenoprotein GPX3 may alleviate renal IRI by inhibiting the MAPK signaling pathway and reducing the expression of NOX, thereby decreasing the release of inflammatory factors (Fig. 8B). Thus, our study identifies selenoprotein GPX3 as a potential new target for alleviating inflammation during renal IRI.

Renal IRI is one of the main causes of AKI, while inflammatory response is an important factor in initiating and exacerbating AKI.^24^ The level of leukocyte infiltration, the production of inflammatory mediators, and the interaction between endothelial cells and leukocytes are significantly increased during the renal IRI-induced inflammatory response.^25^ Neutrophils and macrophages are activated in this process, releasing large amounts of cytokines, ROS, and enzymes, inducing apoptosis and necrosis, and ultimately causing damage to kidney tissue.^25^^,^^26^ The release of pro-inflammatory cytokines and chemokines further promotes leukocyte infiltration and exacerbates the inflammatory cascade.^25^ Current research has demonstrated that inhibiting the inflammatory response is effective in alleviating renal IRI. Scindia et al. found that renal IRI caused significant alterations in systemic iron homeostasis, inducing the onset of an inflammatory response and exacerbating renal tissue damage. In contrast, heparin can alleviate renal IRI by regulating systemic iron homeostasis and reducing the release of inflammatory factors.^27^ Shan et al., on the other hand, found that allicin could improve renal IRI by inhibiting the inflammatory response in rats.^28^ It is thus clear that suppression of the inflammatory response is an important measure in the treatment of renal IRI.

GPX3, a member of the GPX family, is a selenoprotein with antioxidant potential by reducing glutathione and catalyzing hydrogen peroxide, hydroperoxides, and lipid hydroperoxides, thereby controlling the cellular redox microenvironment and preventing oxidative damage.^29^ GPX3 is primarily found in renal tubular epithelial cells, with approximately 70% of GPX3 secreted from the basolateral membrane of proximal tubular cells and released into the plasma through the basolateral artery. Under normal conditions, GPX3 in plasma mainly originates from the kidneys.^30^^,^^31^ However, current studies have found that GPX3 is found in the cytoplasm and plasma membrane of the heart, lung, liver, brain, adipose tissue, mammary gland, and gastrointestinal tract in mammals, in addition to kidney tissue.^30^, ^31^, ^32^, ^33^, ^34^ In addition to the antioxidant properties of selenoprotein GPX3, its anti-inflammatory effects are gradually attracting attention. Reddy et al. found in their study that up-regulation of GPX3 inhibited ROS and hydrogen peroxide production in chronic obstructive pulmonary disease, thereby alleviating the inflammatory response in this disease.^35^ Li et al. found that deficiency of selenoprotein GPX3 increased the expression of the transcription factors NF-κB and HIF1-ɑ, increased the secretion of inflammatory factors, and induced an inflammatory response, which aggravated renal tissue damage.^36^ These findings suggest that GPX3 may be an effective therapeutic target for inhibiting inflammation in tissues and organs. To further explore the role of GPX3 in the inflammatory response during renal IRI, we used classical rat models of renal IRI and cell H/R models. We found that increasing the expression levels of GPX3 could effectively inhibit the secretion of inflammatory factors in renal tissue and cells, alleviating the inflammatory response and thereby protecting against renal IRI.

The NADPH oxidase (NOX) family is a major source of ROS in eukaryotic cells and is likewise important in the regulation of inflammatory responses.^37^^,^^38^ Among them, NOX2 and NOX4 have the highest expression levels in renal tissues.^39^^,^^40^ ROS production is central to the progression of many inflammatory diseases, and ROS are both signaling molecules and inflammatory mediators.^41^ Reducing the expression levels of NOX2 and NOX4 was found to be effective in suppressing the inflammatory response in kidney tissue. Chen et al., in their study of cisplatin-induced AKI, found that NOX2 deficiency was effective in reducing the inflammatory response and alleviating proximal tubular injury.^42^ Li et al. found that inhibition of NOX4 attenuates mitochondrial dysfunction and inflammatory responses and is a potential therapeutic target for septic AKI.^43^ It follows that reducing the expression of NOX oxidase can serve to alleviate the inflammatory response. We found in the present study that when the kidney was subjected to IRI and the cells underwent H/R interventions, the expression levels of both NOX2 and NOX4 were significantly increased, and the secretion of inflammatory factors was significantly increased. Additionally, ROS expression was notably increased in cells, further confirming that NOX oxidases promote the release of inflammatory factors. However, after overexpressing selenoprotein GPX3, the expression levels of NOX2 and NOX4 in renal tissues and cells were significantly reduced, ROS expression was markedly decreased, and the secretion of inflammatory factors was subsequently diminished. These findings indicate that selenoprotein GPX3 can alleviate renal IRI by inhibiting the expression levels of NOX oxidases and reducing the release of inflammatory factors.

The MAPK signaling pathway is a series of highly conserved enzyme-catalyzed reaction cascades that have been closely associated with inflammatory responses.^44^ In our previous study, we found that selenoprotein GPX3 was negatively correlated with the expression of the MAPK signaling pathway during renal IRI using single gene GSEA enrichment analysis, implying that selenoprotein GPX3 could inhibit the activation of the MAPK signaling pathway.^45^ Meanwhile, in our study on selenomethionine alleviation of renal IRI, we demonstrated that inhibition of the MAPK signaling pathway was effective in alleviating the expression levels of inflammatory factors in renal tissues.^20^ In the present study, we found that overexpressing selenoprotein GPX3 in renal tissues and cells significantly suppressed the MAPK signaling pathway and reduced the expression of NOX oxidases, suggesting that NOX oxidase levels are regulated by the MAPK signaling pathway. To further determine whether inhibition of the MAPK signaling pathway was the primary cause of reduced NOX oxidase expression, we used the MAPK signaling pathway agonist anisomycin for intervention. We found that the intervention of anisomycin was able to successfully reverse the inhibitory effect of GPX3 on the MAPK signaling pathway, in which case there was a significant increase in the production of NOX oxidase, an increase in the expression of inflammatory factors, and an increase in the extent of cellular damage. This demonstrates that selenoprotein GPX3 alleviates the inflammatory response by inhibiting the MAPK signaling pathway, which at the same time plays an important regulatory role for NOX oxidase. Therefore, we hypothesized that selenoprotein GPX3 could alleviate the inflammatory response during renal IRI by inhibiting the MAPK signaling pathway and reducing the expression of NOX oxidase.

There are still some limitations in our current study. In the animal experiments, we only detected the expression levels of ALT and AST in the serum of rats after the intervention using AAV9-GPX3, which was used to assess whether the intervention of AAV9-GPX3 caused any damage to liver function. The results showed that there was no statistically significant difference in the expression of ALT and AST in the Sham + GPX3 and IRI + GPX3 groups compared with the Sham and IRI groups (Fig. S1A, B). However, we noticed that there seemed to be a trend toward increased expression levels of AST in the Sham + GPX3 group compared with the Sham group (Fig. S1B). This may be because the AAV promoter we used is a cytomegalovirus, not a promoter specific to kidney tissue. This creates the possibility that it may also interact with other tissues or cells in addition to kidney tissue. However, the effects of AAV9-GPX3 on other tissues and organs of rats were not fully evaluated in our present study, and we will also further explore whether the intervention of AAV9-GPX3 caused toxic damage to other tissues and organs in subsequent experiments.

In conclusion, our results suggest that selenoprotein GPX3 can inhibit the inflammatory response during renal IRI and alleviate renal IRI, which may be closely related to the inhibition of the MAPK signaling pathway and the regulation of NADPH oxidase expression. Therefore, this study demonstrates that selenoprotein GPX3 is a potential target for suppressing inflammation and treating renal IRI. This also supports the potential application of selenoprotein GPX3 in inflammatory diseases.

## CRediT authorship contribution statement

**Jun Pei:** Writing – original draft, Validation, Methodology, Conceptualization. **Jie Zhang:** Validation, Methodology. **Chengjun Yu:** Validation. **Jin Luo:** Validation, Formal analysis. **Sheng Wen:** Methodology. **Yi Hua:** Writing – review & editing, Funding acquisition, Conceptualization. **Guanghui Wei:** Writing – review & editing, Funding acquisition, Conceptualization.

## Ethics declaration

The animal study was reported in accordance with ARRIVE guidelines. All experiments were performed in accordance with relevant guidelines and regulations. The animal study was reviewed and approved by the Animal Ethics Committee of the Children's Hospital of Chongqing Medical University. Written informed consent for publication was obtained from all participants.

## Funding

This work was supported by the Chongqing Natural Science Foundation Innovation and Development Joint Fund Project (China) (No. CSTB2024NSCQ-LZX0072), the Program for Youth Innovation in Future Medicine, Chongqing Medical University (China) (No. W0201), and The Joint Medical Research Project of Chongqing Science and Technology Bureau and Health Commission (China) (No. 2024MSXM002).

## Conflict of interests

Guanghui Wei is one of the Editorial Board members of *Genes & Diseases*, but he/she has no involvement in the peer-review of this article and has no access to information regarding its peer-review. The authors declare that the research was conducted in the absence of any commercial or financial relationships that could be construed as a potential conflict of interests.
