# Supplementary material for: Selenoprotein GPX3 regulates NADPH oxidase expression by inhibiting the MAPK signaling pathway and thereby attenuating the inflammatory response in renal ischemia-reperfusion injury
Source: Genes Dis. 2025 Apr 11;13(2):101640. doi: 10.1016/j.gendis.2025.101640 (PMC12765255; doi:10.1016/j.gendis.2025.101640)
Supplement: Multimedia component 1 [file mmc1.docx]

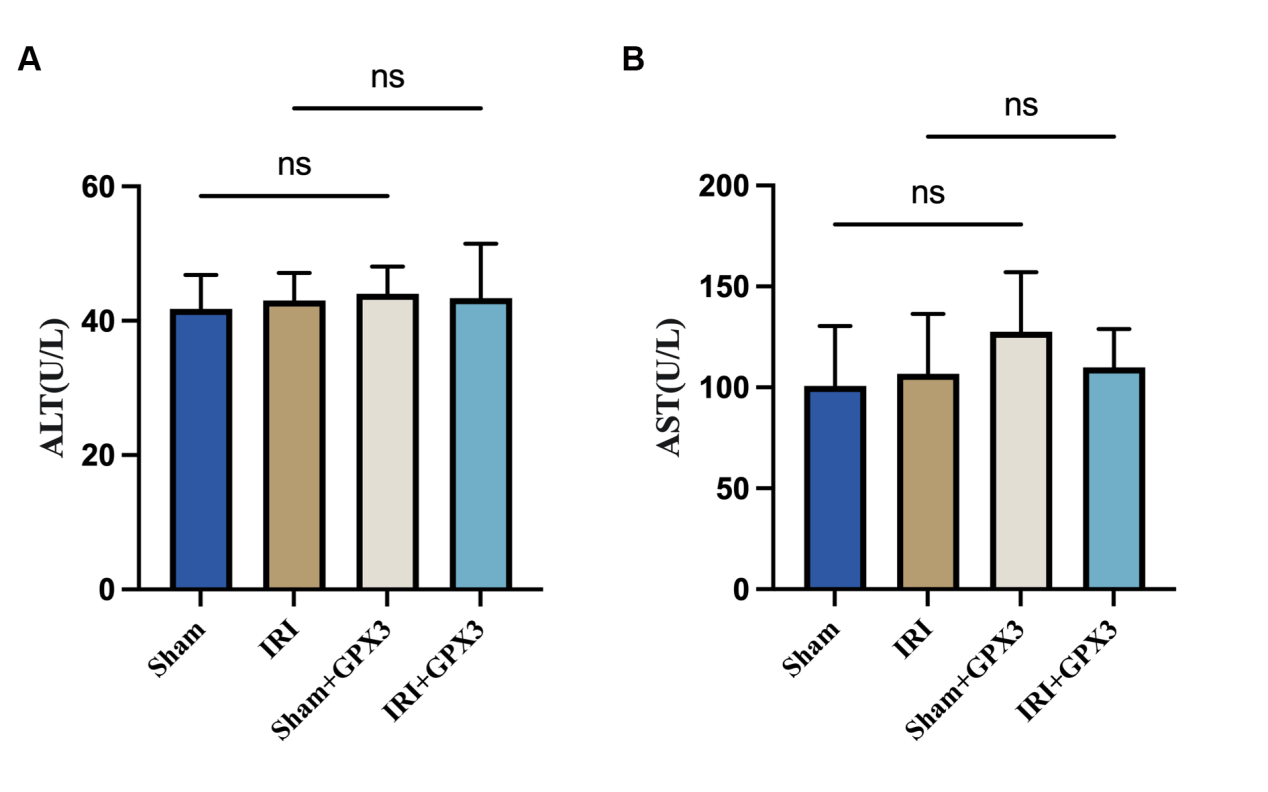


**Supplementary Figure 1. Assays of liver function in rats.** A represents the expression level of ALT in serum of rats in each group; B represents the expression level of AST in serum of rats in each group.
